# Pentacenequinone-Modulated 2D GdSn-PQ Nanosheets as a Fluorescent Probe for the Detection of Enrofloxacin in Biological and Environmental Samples

**DOI:** 10.1021/acsami.4c00277

**Published:** 2024-05-16

**Authors:** Deepak Dabur, Priyanka Rana, Hui-Fen Wu

**Affiliations:** † International PhD Program for Science, National Sun Yat-Sen University, Kaohsiung 80424, Taiwan; ‡ Department of Chemistry, National Sun Yat-Sen University, Kaohsiung, 70, Lien-Hai Road, Kaohsiung 80424, Taiwan; § School of Pharmacy, College of Pharmacy, Kaohsiung Medical University, Kaohsiung 807, Taiwan; ∥ Institute of Medical Science and Technology, College of Medicine, National Sun Yat-Sen University, Kaohsiung 80424, Taiwan; ⊥ Institute of Precision Medicine, College of Medicine, National Sun Yat-Sen University, Kaohsiung 80424, Taiwan; # School of Medicine, College of Medicine, National Sun Yat-Sen University, Kaohsiung 80424, Taiwan; △ Institute of BioPharmaceutical Science, National Sun Yat-Sen University, Kaohsiung 80424, Taiwan

**Keywords:** pentacenequinone, dual fluorescence, bimetallic, 2D nanosheets, enrofloxacin, AIEE

## Abstract

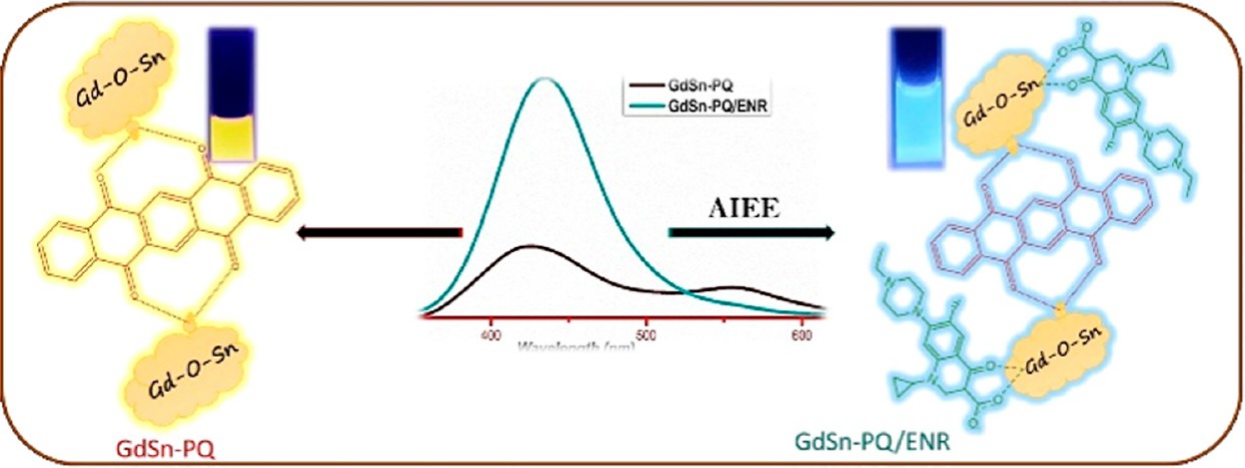

The fate and effects of fluoroquinolone antibacterial (FQ) on the environment are important since there appears to be a surge in FQ resistance like enrofloxacin (ENR) in both environmental and clinical organisms. Numerous reports indicate that the sensing capabilities of these antibiotics need to be improved. Here, we have investigated the interaction of ENR with our synthesized pentacenequinone-modulated gadolinium–tin (GdSn-PQ) nanosheets and the formation of intermolecular interactions that caused the occurrence of aggregation-induced emission enhancement. The concept for designing hybrid metallic nanosheets comes from the unique features inherited from the parent organic precursor. Due to the distinct interaction between ENR and GdSn-PQ, the interstate conversion (ISC) between GdSn-PQ and ENR induces a significant wavelength shift in photoluminescence (PL), improving reliability, selectivity, and visibility compared to quenching- or AIEE-based methods without peak shifts, allowing for highly sensitive and visually detectable analyses. The fluorescence signal of GdSn-PQ exhibited a linear relationship (*R*^2^ = 0.9911), with the added ENR concentrations ranging from 5 to 90 nM, with a detection limit of 0.10 nM. We have demonstrated its potential and wide use in the detection of ENR in biological samples (human urine and blood serum) and environmental samples (tap water and seawater) with a recovery rate of 98– 108%. The current approach has demonstrated that the 2D GdSn-PQ nanosheet is a novel and powerful platform for future biological and environmental studies.

## 1 Introduction

Bimetallization has emerged as a promising strategy to enhance the original single-metal catalyst’s capabilities and produces a new feature, giving it a considerable advantage over monometallic nanoparticles in terms of catalytic performance. The second metal’s inclusion enables the regulation of the catalyst’s activity, selectivity, and stability in certain reactions because of electron density and the length of the metal–metal bond.^1,2^ Due to their unique adjustable optomorphological features derived from delocalized electron networks, two-dimensional (2D) nanostructures based on group IV have recently received a lot of interest. Unidirectional quantum confinement causes the induction of characteristic optical properties in nanosheets or nanoflakes.^3^ Tin-based (Sn) nanomaterials have been intensively investigated for electrochemical analysis, solar energy harvesting, and gas sensing due to certain inherent features such as spin–orbit coupling.^4−6^ Due to distinctive photophysical characteristics, such as strong emission peaks, stable energy levels, a substantial Stokes shift, and great photostability, lanthanide ions are frequently employed to create luminous materials. By combining optoelectronic and magnetic capabilities, rare earth elements exhibit a distinctively strong emission owing to 4f electrons in RE ions and improve the operation of devices.^7,8^ Gadolinium (Gd) may alter the structure’s optical, luminescent, and magnetic characteristics because holes are more active than electrons in Gd 4f states. Adding Gd ions to the Sn lattice increases the conductivity of the holes.^9,10^ Since Gd-based substances are inherently cytotoxic because they may inhibit cell growth and trigger apoptosis, they have been investigated as potential treatments.^11^ These effects are especially helpful in controlling the spread of cancerous cells and bacterial cells that do not react to apoptotic triggers.

Organic precursors play an important role in the synthesis of nanomaterials, facilitating molecular mixing of metals, preventing salt loss, and increasing solution homogeneity. Metals require additional active sites to form symmetrical nanomaterials with high purity and reactivity, which are offered by organic precursors and can generate new possibilities for the production of 2D bimetallic nanosheets.^12,13^ We have used pentacene-5,7:12,14-diquinone (PQ) as the precursor to assemble the 2D bimetallic nanosheets using the probe ultrasonication approach. Pentacene-5,7:12,14-diquinone (PQ) is a rigid, planar molecule with a predisposition to form structured thin films; it is a potential candidate for the formation of bimetallic nanosheets. This pentacene derivative’s ability to interact with metals was found to be due to the presence of aromatic rings and ketone groups, which make it a donor–acceptor–donor (sandwich type) system. Additionally, there are certain advantages to hybrid materials over pure inorganic or organic luminous compounds. Different channels of hybrid materials may adsorb guest molecules of various sizes, achieving very selective and highly sensitive substrate detection. Fluorescent two-dimensional (2D) nanomaterials can be extremely useful for biomedical applications to function as selective probes for designing biological sensors.^14^

By keeping all these in mind, here, we report the unique optical, structural, and antibacterial properties of GdSn-PQ nanosheets synthesized using the probe ultrasonication (PUS) method for antibiotic detection. PUS allows the development of porous materials and nanostructures, as well as the rapid dispersion of chemicals.^12,15^ Antibiotics are interesting because of how often they are used in both human and veterinary medicine, as well as their tendency to contaminate the environment. Enrofloxacin (ENR) is primarily used to treat and prevent bacterial infections in both humans and animals. Some of the ENR will contaminate the ecosystem by entering the soil and water systems through waste. Residential, municipal, and hospital wastewater contain the highest residues of enrofloxacin, with concentrations reaching up to 100 μg/L.^16^ Consequently, there is a considerable concern about the toxicity of enrofloxacin to the ecological environment as well as its effects on the environment. Through the ecological cycle, the misuse of enrofloxacin in aquaculture has a detrimental effect on the aquatic ecological environment and may have an effect on public health.^17^ Therefore, it is crucial to provide a quick and easy method to measure the ENR content in different water bodies. However, their improper usage has resulted in many severe undesirable side effects, including headache, sleeplessness, hematuria, nausea, vomiting, and diarrhea. This poses a risk to the public’s health. The highly sensitive and selective detection methods of antibiotics, which include surface-enhanced Raman scattering (SERS),^18^ mass spectrometry (MS),^19^ high-performance liquid chromatography (HPLC),^20,21^ spectrophotometric techniques,^22^ gas chromatography,^23^ voltammetry,^24^ immunoassays,^25^ electrochemical sensors,^26^ colorimetric detection,^27^ and fluorescent sensors,^28,29^ have been intensively used to prevent problems with human health caused by residual. However, the pretreatment processes for these approaches are limited and have many drawbacks such as time-consuming procedures, labor-intensive protocols, and the need for expensive instrumentation, making them unsuitable for routine analysis. In contrast, fluorescent sensors based on various novel nanomaterials, especially 2D nanosheets,^15,30,31^ have drawn a lot of interest because they can effectively reduce the effects of changing local environmental conditions, such as variations in the concentration of the probe. However, mostly, fluorescence sensors are based on monochromatic fluorescence change, despite the creation of numerous chemical sensors based on the alteration of fluorescence brought about by the interaction of a sensor with an analyte.^32^ However, a molecule that can alter its multicolor fluorescence in response to environmental factors makes it ideal for sensing with only the human eye because it can quickly identify and detect analyte concentration without the use of numerous analytical tools. Additionally, multicolor fluorescence sensors can be used to detect molecule aggregation,^32^ intramolecular hydrogen bonding, and excited-state proton transfer.^33^ Therefore, there is a great potential to increase precision, sensitivity, and selectivity for detection using the 2D GdSn-PQ nanosheets due to their dual emission properties. The dual emission GdSn-PQ nanosheets were initially used in this study, utilizing a one-pot solvothermal technique using pentacenetetrone as the precursor and THF as the solvent. The entire preparation procedure is easy, quick, and free of additional harmful reducing chemicals. A significant new emission peak with ENR is visible in the produced nanosheets through the aggregation-induced enhancement emission aggregation-induced emission enhancement (AIEE). The interstate conversion (ISC) between the GdSn-PQ nanosheets and the ENR stimulated photoluminescence with a considerable wavelength shift. Compared to previous methods that relied on quenching or AIEE without peak shifts, this phenomenon/detection improves reliability and visibility. The distinctiveness stems from the unique interaction between enrofloxacin and nanosheets, which causes a discernible alteration in emission spectra, allowing for sensitive and visually detectable analysis. Therefore, for the first time, we have demonstrated on both environmental and biological sensing of ENR by using 2D GdSn-PQ nanosheets.

## 2 Experimental Section

### 2.1 Synthesis of the Pentacene 5,7:12,14 Diquinone (PQ)

First, 2-methyl 1,4-naphthoquinone (0.6 g) was added to 50 mL of ethanol and stirred to create a homogeneous solution. Then, 1 mL of *N*-methylcyclohexylamine was added dropwise into the above solution. The solution was then left in the dark overnight without stirring. A light brown product was produced after filtering. Pentacene-5,7:12,14-diquinone (PQ) was produced by the recrystallization of the brown product with chloroform.^34^

### 2.2 Preparation of 2D GdSn-PQ Nanosheets

**Figure 1:**
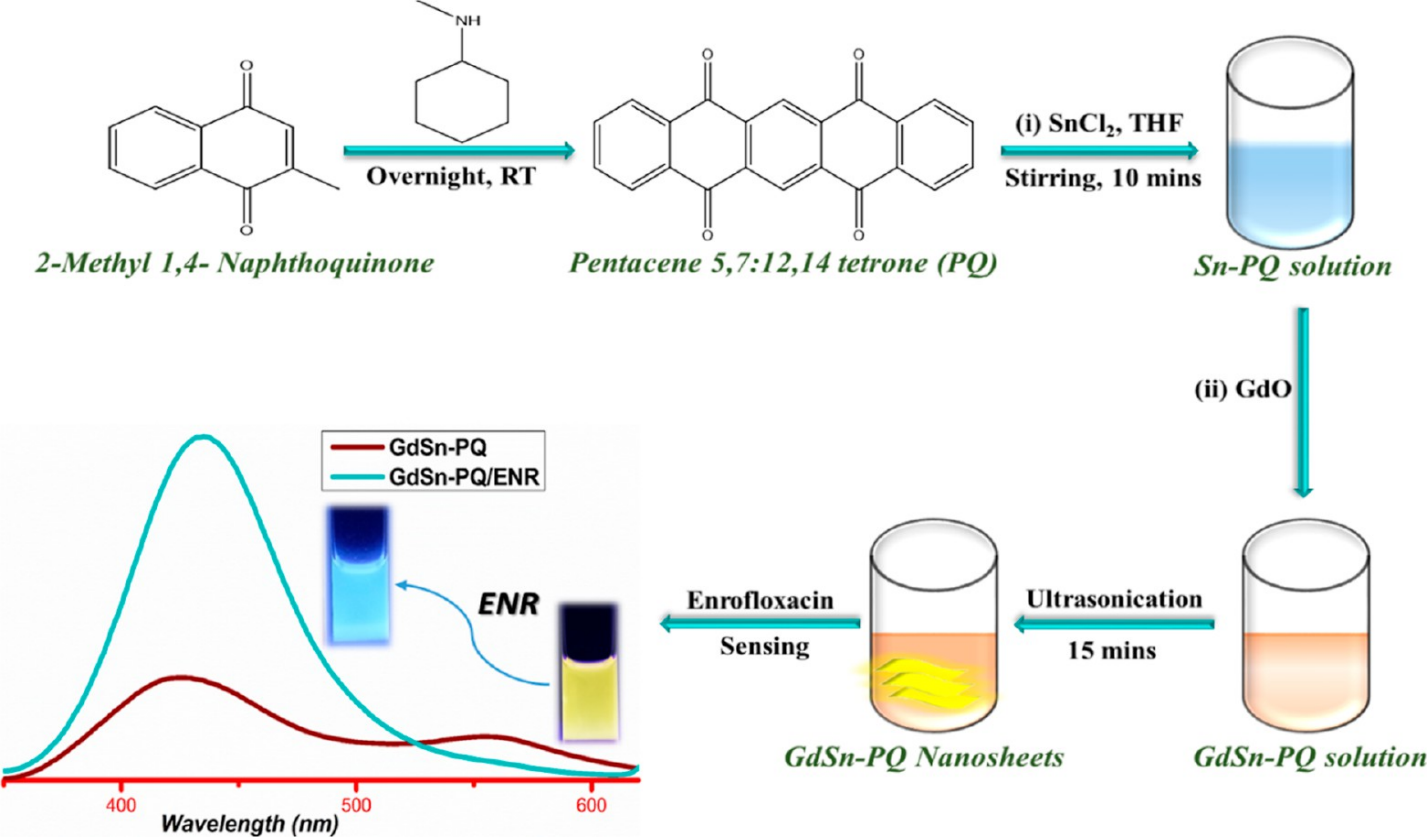
Schematic representation showing the procedures for the synthesis and preparation of the GdSn-PQ nanosheets and their application in ENR detection.

To create a homogeneous solution, 10 mL of tetrahydrofuran (THF) with 5 mg of PQ was agitated for 10 min. Then, 10 mL of the aforementioned solution containing 1:3 mol of Gd:Sn with 10 mg of SnCl_2_ and Gd_2_O_3_ was added. The ultrasonication probe (PUS) was then immersed in the solution for 15 min (2 s ON and 1 s OFF). The bimetallic nanosheets shown in Figure 1 were obtained after a further 2 h of incubation. Furthermore, nanosheets were characterized and applied for sensing purposes, as shown in Figure 1. Some controlled experiments were also performed based on different Gd:Sn mole ratios in PQ solution to achieve the dual fluorescent nanosheets as shown in Figure S1a.

### 2.3 Procedure for the AIEE Sensing of Enrofloxacin and Selectivity

ENR was spiked into PBS buffer (0.01 M, pH 7.4) at a range of concentrations from 5 nM to 90 nM. After that, 100 μL of GdSn-PQ was added to every spiked solution and gently shaken for 2 min. The spectra of fluorescence emission were captured at 320 nm excitation. One mM of spectinomycin (SPM), lomefloxacin (LFX), ampicillin (AMP), and ciprofloxacin (CIP) were chosen as competing compounds that were structurally comparable to ENR (1 mM) in order to evaluate the selectivity of GdSn-PQ. The studies involved adding 100 μL of GdSn-PQ to the antibiotic solutions (1 mM) and monitoring the changes in the fluorescence signal.

### 2.4 Interference Study for Probing the Photoluminescence Stability of 2D GdSn-PQ

A series of tests based on the suspension of GdSn-PQ were thus carried out to explore the application in actual biological and environmental samples and see if it could identify antibiotics in urine as well. Human urine is mostly composed of uric acid, NaCl, KCl, NH_4_Cl, Na_2_SO_4_, glucose, and urea, which have been prepared for detection. To test the anti-interference capabilities of ENR in GdSn-PQ aggregation fluorescence sensing, interferences were used as interference items.

### 2.5 Real Sample Assay (Biological Perspective)

Fresh, filtered urine was used to prepare the stock solution of ENR, to which four different concentrations of ENR (5, 10, 20, and 30 nM) were added with 100 μL of GdSn-PQ. The mixture was then diluted with PBS to a final volume of 1 mL. At 320 nm excitation, emission values were also taken (*n* = 3). The same experiment was also carried out using a blood serum sample. Three replicates were done using every concentration (*n* = 3). Urine samples were collected with the donor consent for all experiments, and approval from the ethical committee at the National Sun Yat-sen University in Taiwan was obtained. The commercial bovine blood serum was spiked with GdSn-PQ for this study.

### 2.6 Collection and Analysis of Water Samples (Environmental Perspective)

In addition to sampling seawater from Kaohsiung, Taiwan’s Siziwan Bay, tap water was collected from our lab. After centrifuging, a 0.22 m membrane was used to filter each sample. The samples were subsequently treated with a buffer solution to bring their pH levels to 6.59. Before detection, the samples were kept at 4 °C. Different amounts of ENR stock solution (5, 10, 20, and 30 nM) were added to tap water and seawater samples, and the spiked sample solutions were then subjected to fluorescence (*n* = 3). Three replicates were done using every concentration.

## 3 Results and Discussion

### 3.1 Synthesis and Optical Characterizations

**Figure 2:**
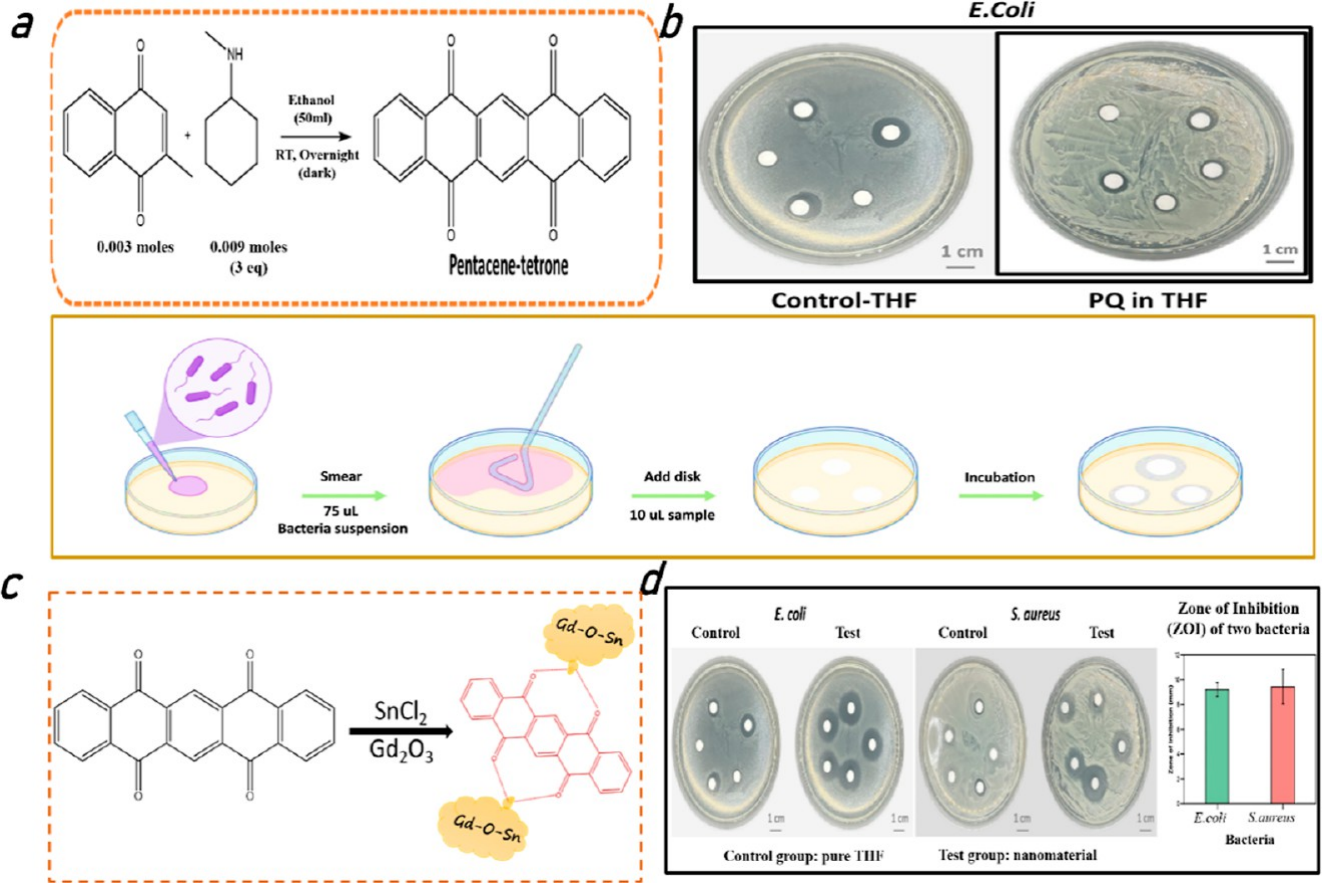
(a,c) Synthesis routes and (b,d) antibacterial activity test for the PQ precursor and the GdSn-PQ nanosheets.

First, using the Suzuki–Miyaura coupling process, we have synthesized pentacenetetrone (PQ) (Figure 2a), which serves as the organic scaffold for the preparation of GdSn-PQ nanosheets, as shown in Figure 2c. PQ was confirmed using Fourier transform infrared (FTIR)analysis, nuclear magnetic resonance (NMR), and UV absorption, as shown in Figure S1. FTIR spectroscopy: (704, 969, 1128, 1266, 1318, 1591, 1668 cm^–1^) and ^1^H NMR (400 MHz, CDCl_3_): 9.25 (2H, s), 8.40 (dd, 4H), 7.85 (dd, 4H), as shown in Figure S1c,b, respectively.^35,36^ The inhibitory effect was tested against *E. coli* and *S. aureus* to determine the impact of delamination on the antibacterial effectiveness of PQ and GdSn-PQ nanosheets. Photos of agar plates on which control (THF) and bacterial cells were recultivated after being treated for 4 h with a concentration of 100 μg/mL of PQ and GdSn-PQ are shown in Figure 2b,d, respectively. As shown in Figure 2b, PQ shows a very blank behavior against *E. coli,* but GdSn-PQ shows good antibacterial activity against both *E. coli* and *S. aureus*. The growth suppression of both bacterial strains exposed to the GdSn-PQ nanosheets under investigation is also shown in Figure 2d. *E. coli* and *S. aureus* vitality decreases, showing higher inhibition, to 9 ± 0.5 mm. The GdSn-PQ nanosheets’ potent antibacterial properties may in part be explained by the anionic nature of their surface because of the presence of electron-rich metal ions. The anionic nature is proved by the zeta potential in later sections.

**Figure 3:**
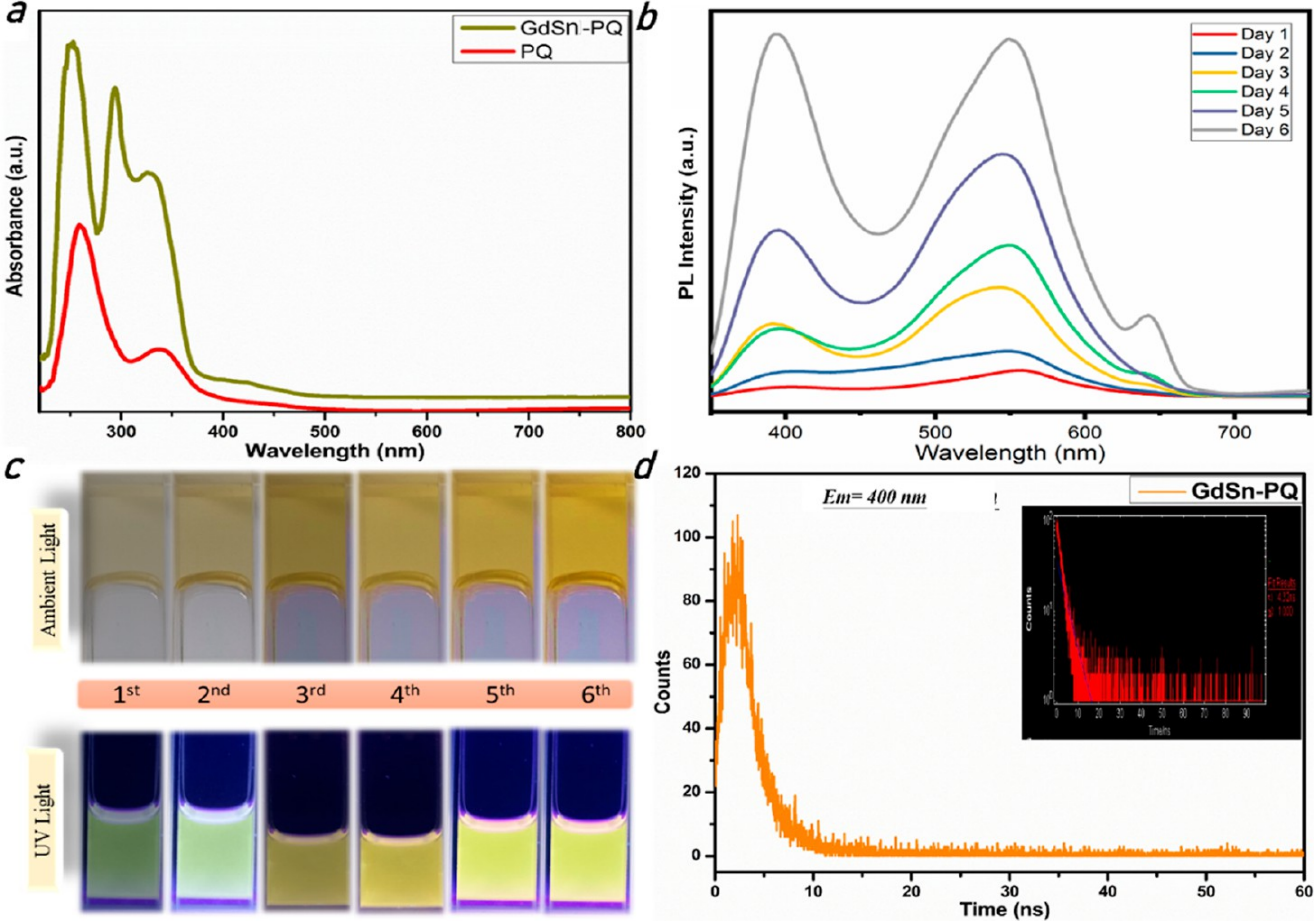
Optical properties of GdSn-PQ nanosheets: (a) UV comparison of GdSn-PQ and PQ, (b) recorded PL emission from GdSn-PQ for 6 consecutive days, (c) ambient light (upper panel) and UV-illuminated (lower panel) images of the GdSn-PQ nanosheets shown for 6 days, and (d) time-resolved photoluminescence (TRPL) graph for λ_em_ at 400 nm.

Additionally, the optical characteristics of GdSn-PQ nanosheets were studied in Figure 3. Both PQ and GdSn-PQ displayed several UV–vis absorption peaks, as shown in Figure 3a, but GdSn-PQ also had a stronger UV–vis characteristic absorption peak at 300 nm because of the formation of the interaction of Gd–Sn metals with the PQ moiety. PQ has a wide spectrum at 260 nm, which is consistent with the presence of the pentacene moiety, and the highest occupied molecular orbital–lowest unoccupied molecular orbital (HOMO–LUMO) transition primary absorption characteristics were seen at 340 nm. GdSn-PQ has three absorption peaks between 250 and 350 nm. Further, the shift in the pentacene moiety peak from 260 to 250 nm may be due to the presence of Sn.^37^ The peak present in GdSn-PQ at 290 nm and the shift of the absorption peak from 340 nm to 325 nm confirmed the presence of Gd metal in the structure.^38^ The dual peaks at 400 and 550 nm are seen in the fluorescence investigation of GdSn-PQ in Figure 3b, which shows that the yellow emission of GdSn-PQ is caused by the different intensity ratios of both peaks. For 6 days straight, PL emission was monitored to check the stability of fluorescence.

Figure 3c depicts the visible fluorescence; the upper panel displays photographs taken in ambient light from the 1st to the 6th day of synthesis, while the lower panel displays images taken under UV irradiation of the same synthesized samples. Figure S2 demonstrates the comparison of the PL emission of synthesized GdSn-PQ and PQ. Another accomplishment of the work is achieving steady and constant emissions from the GdSn-PQ nanosheets made at ambient temperature. Finally, the significant peaks at 400 nm observed in Figure 3d are subjected to time-resolved photoluminescence (TRPL) measurements. The observed relaxation time is 4.32 ns, retaining χ^2^ unity.

### 3.2 Structural Characterizations of 2D GdSn-PQ

**Figure 4:**
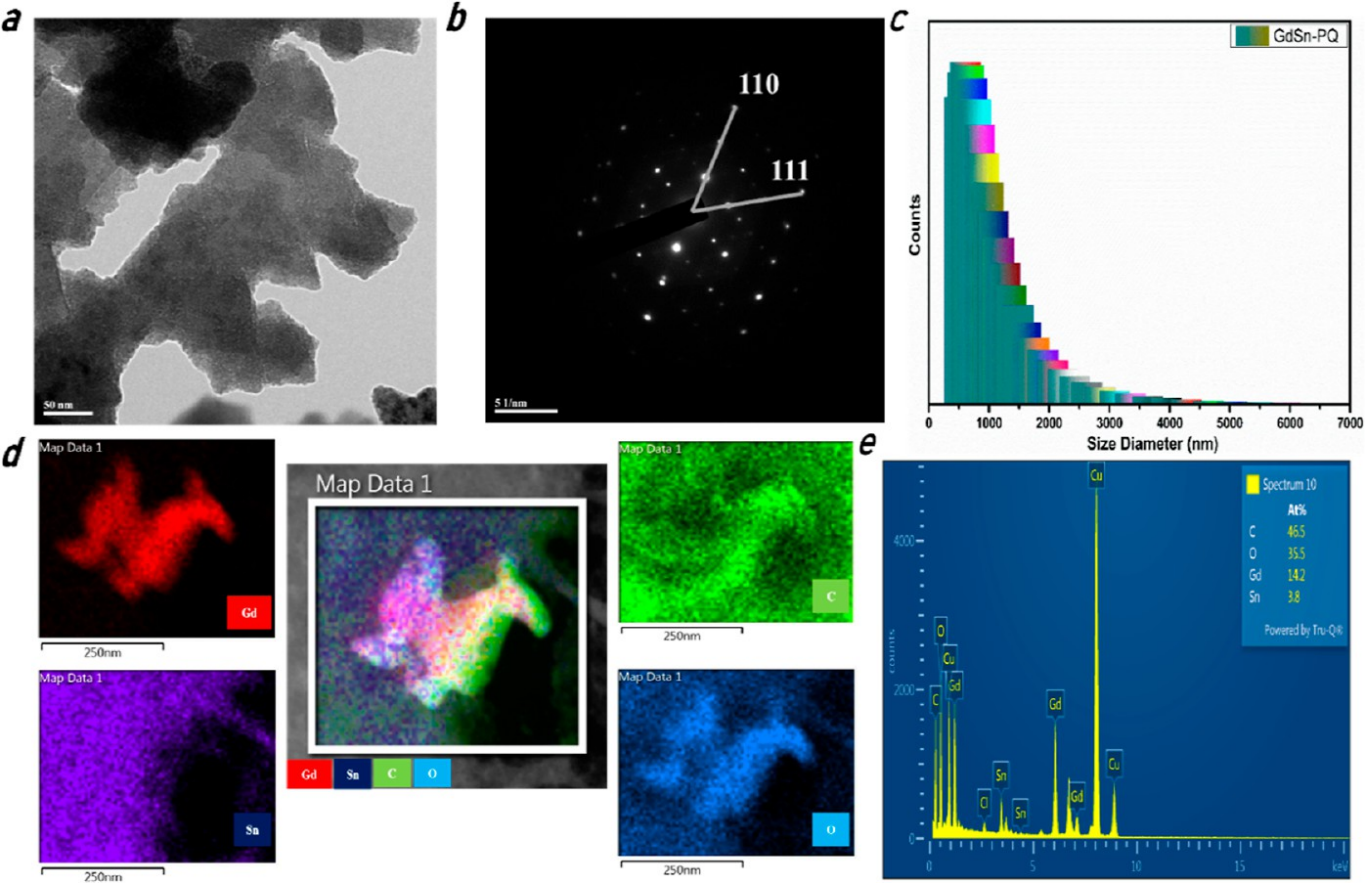
Structural characterization of the GdSn-PQ nanosheets: (a) TEM image, (b) SAED pattern, (c) DLS size graph, (d) high-angle annular dark-field scanning transmission electron microscopy image elemental mappings of GdSn-PQ: C (green color), Gd (red color), O (blue color), and Sn (violet color) and single element mapping of C (green color), O (blue color), Gd (red color), and Sn (violet color). (d) Elemental ratios shown in the EDS pattern.

The structural and elemental composition of 2D GdSn-PQ nanosheets is described in Figure 4. Layered nanosheets were seen in the transmission electron microscopy (TEM) images in Figures 4a and S3a,b. The TEM images demonstrate the existence of two-dimensional nanostructures with a range of thicknesses. Properly aligned nanosheets with the size range of 350–600 nm on 50 nm (Figure 4a), 20 nm (Figure S3a), and 100 nm (Figure S3b) scale are observed in GdSn-PQ. The selected-area electron diffraction (SAED) pattern clearly shows the polycrystalline nature of GdSn-PQ nanosheets (Figure 4b). The SAED analysis of GdSn-PQ displays interlayered hexagonal diffraction, as shown in Figure 4b. The modification persists in the tetravalent nature of Sn, achieving a honeycomb lattice. The prominent diffraction occurs at planes (111) and (110), which are the widely acknowledged hexagonal Sn planes.^60^ The interspacing difference visible in polycrystalline layers is due to the presence of Gd defects in the planar lattices. These planes are also observed in the grazing incidence X-ray diffraction analysis of the sample shown in Figure S3c. The crystal orientations observed at 2θ values are [111] at 28.58, [100] at 33.17, and [110] at 47.4, respectively. These facets are indicative of a hexagonal Sn nanostructure with Gd^2+^ orientations at primitive points. With the diameters ranging from 500 to 1000 nm, dynamic light scattering (DLS) reveals the good dispersion of the GdSn-PQ nanosheets (Figure 4c) in the solution. The chemical composition of GdSn-PQ (C, O, Gd, and Sn) was confirmed by elemental mapping (Figure 4d) and energy-dispersive spectroscopy (EDS) in Figure 4e, with Gd and Sn being evenly distributed across the whole GdSn-PQ nanosheets.

The high-angle annular dark-field scanning transmission electron microscopy (HAADF-STEM) pictures in Figure 4d depict the elemental distribution in GdSn-PQ nanosheets: red denotes Gd metal, violet denotes Sn, green denotes C, and blue denotes O. EDS (Figure 4e) validated the elemental ratios of the nanosheets, which showed the C/O/Gd/Sn ratio of 12.2:9.34:3.7:1, respectively.

**Figure 5:**
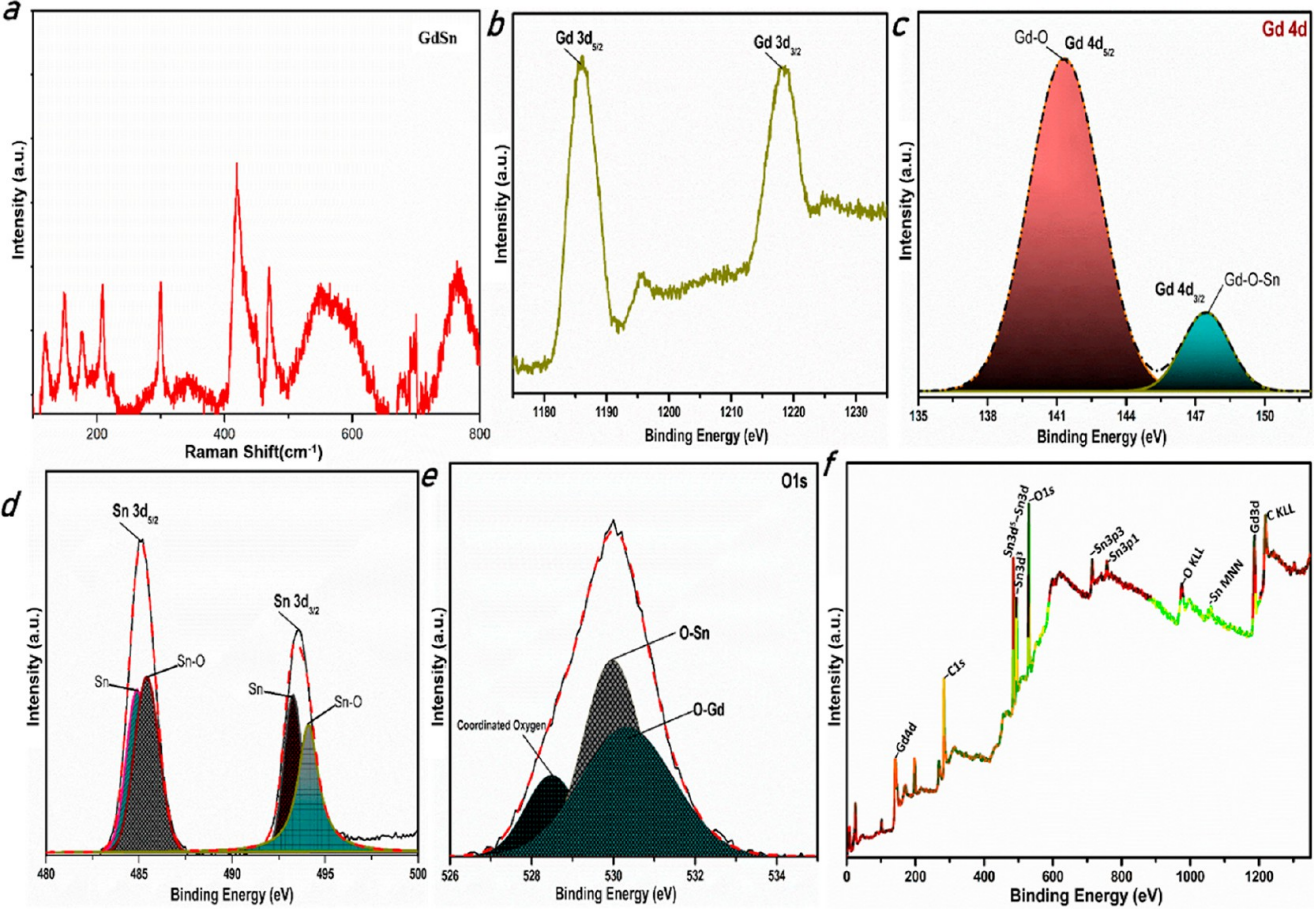
Bonding analysis of the GdSn-PQ nanosheets: (a) Raman spectra. XPS spectrum of (b) fitted Gd 3d with spin–orbit splitting of Gd 3d_3/2_ and Gd 3d_5/2_, (c) fitted Gd 4d with spin–orbit splitting of Gd 4d_3/2_ and Gd 4d_5/2_, (d) fitted Sn 3d with spin–orbit splitting of Sn 3d_3/2_ and Sn 3d_5/2_, (e) fitted O 1s spectra with coordinated oxygen bonds with metals, and (f) survey scan spectra.

There are distinct peaks in the Raman spectra of GdSn-PQ films (Figure 5a). Notably, a peak at 113 cm^–1^ (B_1g_) and that at 210 cm^–1^ (A_1g_) show the existence of Sn–O bonds in the structures.^39,40^ In addition, we find acute peaks at 300 and 410 cm^–1^, as well as a broad peak at 545 cm^–1^, which are related to O–Gd–O stretching bands.^41^ There is a slight shift in the peak wavelength because of the presence of Sn bonds. Raman peaks between 650 and 800 cm^–1^ are associated with A_1g_ and B_2g_ and are indicative of the presence of O–Sn–O bonds in the structure.^42^ The pentacene moiety may possibly be responsible for the lower wavelength peaks of 100–200 cm^–1^. X-ray photoelectron spectroscopy (XPS) is also used to look at the chemical composition of the synthesized GdSn-PQ nanosheets. Figure 5b displays the Gd 3d high-resolution XPS spectrum. The two significant peaks at 1187 and 1220 eV, which correspond to a spin–orbit splitting of 32 eV, are the 3d_5/2_ and 3d_3/2_ energy levels of Gd, respectively.^43,44^ To explore the bonding possibilities of Gd, we have studied the Gd 4d XPS spectrum in Figure 5c. The two subpeaks of Gd 4d at 141.3 and 147.2 eV suggest that the chemical state of the Gd element in the film is composed of two types, which are Gd–O and Gd–O–Sn, respectively,^45^ rather than the pure form since the binding energy is 144 eV.^46^Figure 5d shows the spectrum of the Sn 3d core level, which has two pairs of peaks between 484 and 495 eV. Peaks at 485.5 and 494 eV confirm the presence of Sn–O bonds in the structure; these two peaks are attributed to the spin–orbits of Sn 3d_5/2_ and Sn 3d_3/2_, respectively, with a splitting of 8.50 eV.^47−49^ However, the Sn 3d_5/2_ and Sn 3d_3/2_ peaks exhibit a shift in binding energies, which may be related to the oxygen deficiency that can be filled by Gd ions. The peaks at 484.8 and 493.3 eV may be due to the metallic Sn bonds.^50^Figure 5e shows that the two major peaks at 529.7 and 530.4 eV are due to the presence of metallic oxygen bonds O–Sn^47,51^ and O–Gd.^52^ A small shoulder with the linearly coordinated oxygen appears at 528.4 eV^53^ due to the coordination bonds between C–O–metal and metal–O–metal.

The survey XPS spectrum is shown in Figure 5f, and all of the peaks may be attributed to electronic transitions in Gd, Sn, O, and C.

Figure S4 shows the C 1s spectra with all the possible bonds (C=C and C–C from PQ, and C–O) and peaks present in structures^54−56^ and confirms the breaking of C=O bonds from the PQ moiety and the formation of new bonds with metal ions.

### 3.3 Application of 2D GdSn-PQ for ENR Detection

**Figure 6:**
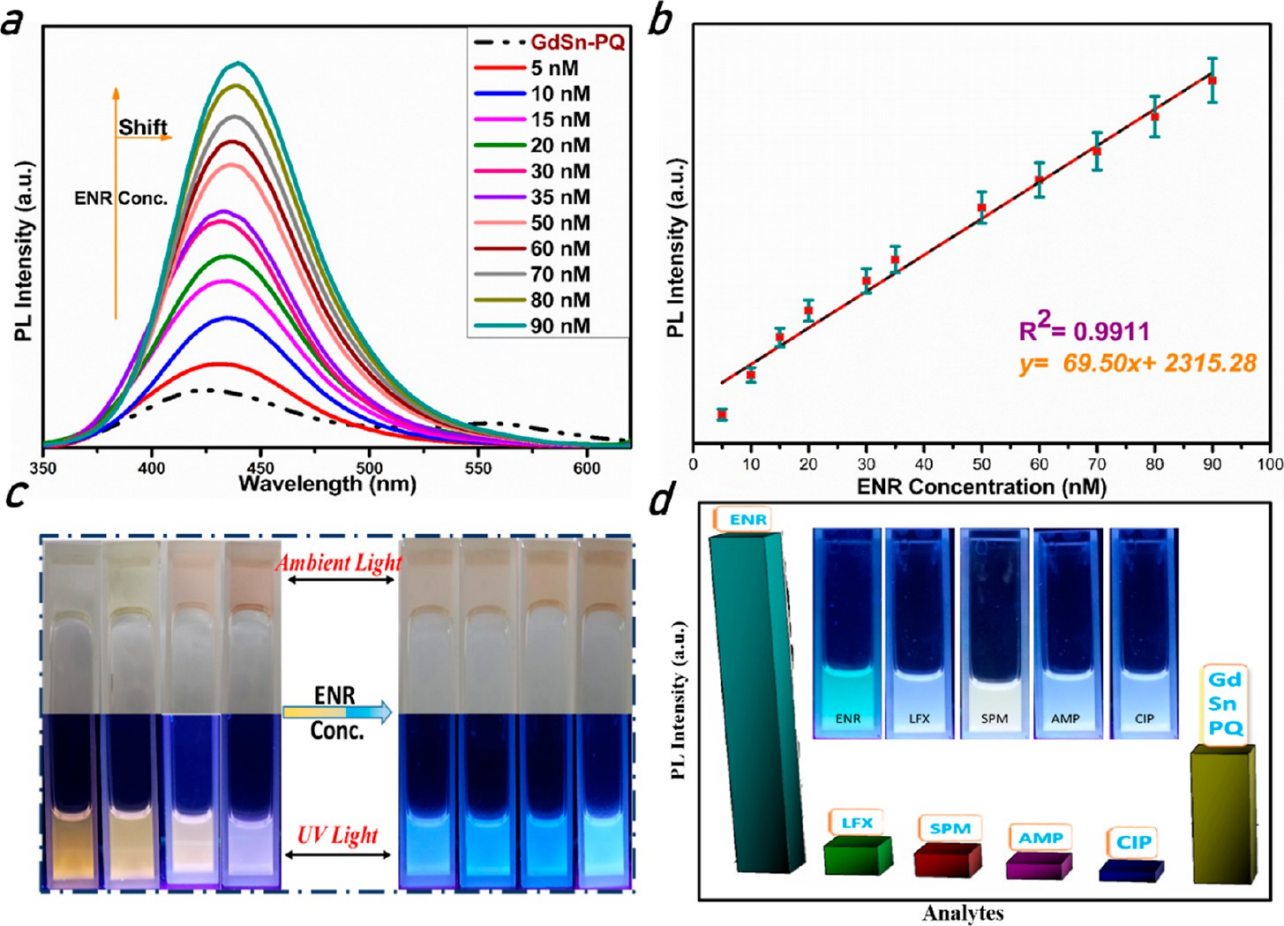
Sensor studies for ENR detection: (a) different concentration studies of ENR detection, (b) calibration curves of ENR plotted using the Stern–Volmer graph (*n* = 3), (c) ambient light (upper panel) and UV-illuminated (lower panel) images of the GdSn-PQ nanosheets after the addition of ENR concentrations, and (d) specificity experiment of GdSn-PQ for the detection of ENR. ENR (1 mM) and the other four antibiotics (1 mM) were used in the specificity experiments.

Different fluorescence spectra were produced by varying the concentration of ENR in order to assess the sensitivity of the fluorescent probe based on the GdSn-PQ nanosheets under ideal circumstances. The detection system was created by combining 100 μL of GdSn-PQ with various ENR concentrations. For a linear investigation, the fluorescence signal of the entire system was measured at an excitation wavelength of 320 nm. The fluorescence of the system demonstrated a considerable shift in both emission peaks with increasing ENR addition, demonstrating a positive relationship between the fluorescence intensity of the whole system and ENR addition in the range of 5–90 nM (Figure 6a). After the ENR was applied, the aggregation effect caused both emission peaks to combine to create a new emission peak at 435 nm with a blue shift.

As a result, there was a strong linear correlation between the concentration of ENR and the level of fluorescence amplification. In this instance, the correlation coefficient (*R*^2^) was 0.9911, and the regression equation was *y* = 69.50*x* + 2315.28 (Figure 6b). According to the limit of detection (LOD) calculations presented in the Supporting Information, the detection limit (3.3 × SD/*S*) is calculated to be 0.10 nM. The concentration-based study of ENR can also be seen in Figure 6c, where we have shown the change in color based on the concentration in ambient light (upper panel) and UV light (lower panel). This whole experiment for the detection of ENR was concluded within 10 min after the mixing of GdSn-PQ and ENR, revealing that the time needed for the fluorescence sensor to be formed and functioning was very short. Other classical antibiotics, including ciprofloxacin (CIP), spectinomycin (SPM), lomefloxacin (LFX), and ampicillin (AMP), were chosen for the specificity study (chemical structures of all antibiotics are shown in Figure S5). Surprisingly, the overall fluorescence intensity of GdSn-PQ was quenched by the addition of a high dosage of these antibiotics except ENR, as shown in Figure 6d. The UV-illuminated images corresponding to all antibiotics after adding GdSn-PQ nanosheets are shown in Figure 6d. When assessing PBS spiked with ENR (1 mM) and other widely used antibiotics (1 mM), GdSn-PQ demonstrated good specificity.

### 3.4 Interference Studies

**Figure 7:**
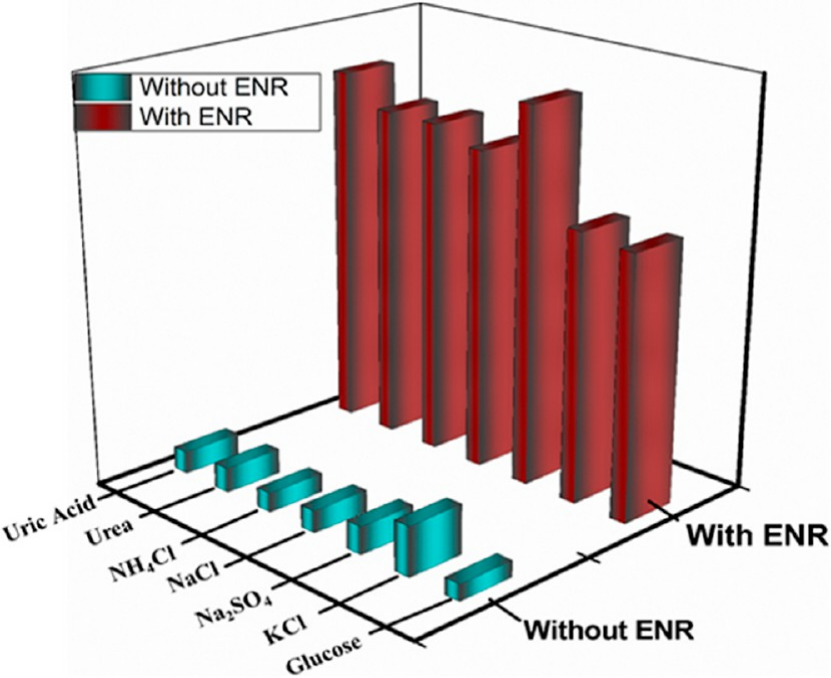
Interference (concentration = 1 mM) studies of different urine components in a solution with the GdSn-PQ nanosheets.

The applicability of GdSn-PQ nanosheets for the detection of ENR in environmental applications as well as biological fluids (human urine and blood serum) was investigated due to their strong fluorescence characteristics, high stability, and selectivity toward ENR. In order to find out if it could detect antibiotics in urine, a number of tests based on the suspension of GdSn-PQ were carried out. The essential elements in human urine are NaCl, KCl, NH_4_Cl, Na_2_SO_4_, glucose, and uric acid. GdSn-PQ demonstrated strong anti-interference capacity when ENR was identified in an aqueous solution because its fluorescence intensity decreased when other components in urine were added, whereas it increased quickly when ENR was added to the solution (Figure 7).

### 3.5 Biological Sample Assay of Human Urine and Blood Serum

**Figure 8:**
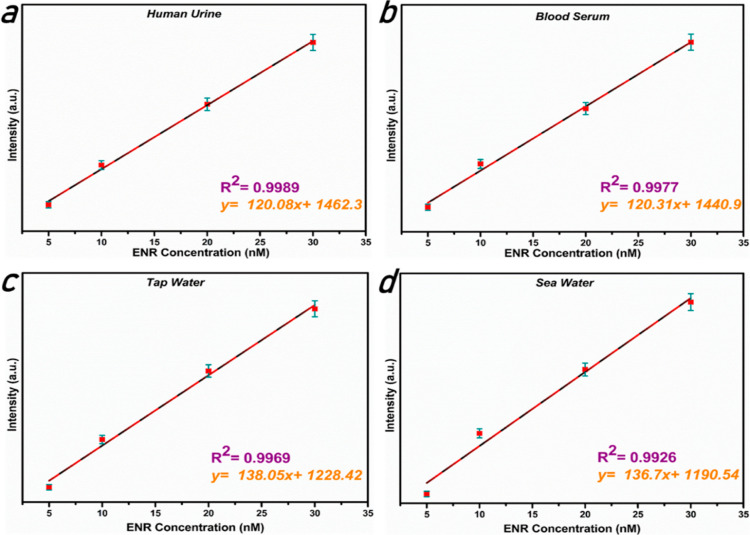
Biological and environmental real sample studies (the Stern–Volmer curves) in (a) human urine, (b) blood serum, (c) tap water, and (d) seawater samples. Three replicates were done for all real samples (*n* = 3).

ENR is most frequently used to treat and prevent bacterial infections in people and animals. Mammals have an elimination half-life for ENR that is between 1.2 and 3.3 h shorter than that of chicken, and about 40% of fluoroquinolone remains linked to plasma proteins.^57^ Monitoring ENR levels in blood serum may thus be crucial to controlling antibiotic levels. Blood serum and urine were spiked with ENR at doses of 5, 10, 20, and 30 nM, and they were filtered through 0.2 m syringe filters before having the pH adjusted to 7.4. Fluorescence emission was detected when GdSn-PQ (100 μL) was introduced to the various ENR concentrations prepared in biological fluids. The outcome demonstrated that the response was linear in the 5–30 nM range, with *R*^2^ = 0.9989 for human urine (Figure 8a) and 0.9977 for blood serum (Figure 8b), and the LOD was found to be 0.26 nM for biological fluids using the Stern–Volmer graph. The corresponding PL data are shown in Figure S6a,b for human urine and blood serum, respectively. The recovery findings for blood serum and urine samples are also displayed in Table 1. The suggested approach produced ENR recoveries in biological fluids that varied from 98.0 to 109.0% on average.

**Table 1:** Recovery Results of ENR in Different Real-World Samples

		concentration added (nM)	concentration detected (nM)	recovery (%)	RSD (%) (*n* = 3)
biological	human urine	5	5.24	104.78	2.43
		10	10.92	109.19	2.47
		20	20.08	100.41	2.94
		30	29.87	99.56	0.92
	blood serum	5	4.92	98.35	4.25
		10	10.81	108.11	5.47
		20	19.80	99.03	1.99
		30	29.98	99.92	0.68
environmental	tap water	5	4.90	98.18	4.21
		10	10.88	108.84	1.68
		20	21.27	106.37	2.70
		30	30.98	103.27	2.13
	seawater	5	4.89	98.01	4.19
		10	10.92	109.16	5.49
		20	20.63	103.13	1.94
		30	30.76	102.53	1.10

### 3.6 Environmental Sample Assay for Tap Water and Seawater

GdSn-PQ was used to find ENR in seawater and tap water in order to investigate the applicability of the approach to environmental samples. Seawater and city water were used to make ENR stock solutions. In order to conduct fluorescence experiments, four different doses (5, 10, 20, and 30 nM) of the previously generated ENR stock solution were combined with GdSn-PQ nanosheets. A 0.2 m syringe filter was used to filter out any macro-sized particles in water from each of the spiked solutions. For each concentration, the test was carried out three times. With the regression values of *R*^2^ = 0.9969 and *R*^2^ = 0.9926 for tap water and seawater, respectively, the findings demonstrated a linear response from 5 to 30 nM (Figure 8c,d) with a higher detection limit (LOD) value of 0.30 nM. GdSn-PQ nanosheets show great recoveries for environmental samples ranging from 98 to 109%, as shown in Table 1. The corresponding PL data are shown in Figure S6c,d for tap water and seawater, respectively. One of the key parameters affecting the sensor performance is pH effect, which can affect a detection method’s sensitivity in different matrices for a variety of reasons. The analyte and sensing probes’ interaction can be influenced by pH. The ionization state of functional groups or the surface charge of sensing elements can be changed by pH changes, which can impact the specificity and affinity of the analyte–probe interaction. As a result, there may be variances in response kinetics or signal intensity, which could affect the sensitivity in various matrices. Chemical interferences can also be introduced by pH variations in various matrices, which can impact the sensor performance. For example, basic or acidic substances found in urine, serum, or other water bodies may interact with the sensing elements or obstruct the detection. Understanding and regulating pH conditions are critical for optimizing the sensor performance and ensuring accurate detection across a wide range of samples. Hence, we have studied the PL response of GdSn-PQ in different pH values. The fluorescence data in Figure S7a show that the PL intensity and wavelength of GdSn-PQ nanosheets are consistent across a pH range. This pH independence indicates that the nanosheets are stable under varying environmental conditions, implying that differences in sensitivities observed across matrices are unlikely to be due to pH variations. As a result, GdSn-PQ nanosheets’ stable PL response lends itself to reliable detection applications in a variety of sample matrices. However, pure ENR shows different PL behaviors in different mediums, as shown in Figure S7b, which leads to these discrepancies in results. However, GdSn-PQ exhibits consistent results across various mediums due to the exceptional stability. The developed sensor was compared with the reported sensor using various methods, and the results are provided in Table S1 to demonstrate the sensor capability. The current method in this study also has the advantages of being rapid and convenient, not requiring large instruments and reducing agents when compared with other methods for detecting ENR, such as HPLC, SERS, EC, and ICA. Unlike alternative methods which usually require the incorporation of harmful reducing and oxidizing chemicals to induce specific reactions or modify the materials involved, this approach capitalizes on the inherent properties of the PQ and Gd–Sn interaction without necessitating external additives or high temperatures. This simplicity speeds up the process, reducing the time and resources needed for preparation. PQ can also be prepared without using high temperatures or reducing agents. Furthermore, the lack of harmful reducing agents is a green approach that can reduce the risk of environmental contamination and ensures the safety of those handling the materials. GdSn-PQ has a higher sensitivity for detecting ENR than previous fluorescence sensors, which are typically quenching, and AIEE sensors with no shift in wavelength. Unlike the conventional quenching or AIEE methods, the ISC-mediated peak shift improves the reliability and selectivity of analytical detection. The ISC-induced spectral changes make ENR analysis more sensitive and visually detectable. This change in PL properties is due to the altered electronic states of the GdSn-PQ complex during interaction. Understanding the complexities of this, further investigation into the underlying mechanisms in a later section may reveal novel strategies for improving the detection sensitivity and specificity in pharmaceutical and environmental monitoring. This makes our method to be more suitable and reliable, especially as a naked eye sensor. The current ENR sensing system has a wide detection range, a low LOD, and is applicable to both environmental and biological fields. We have created an ultrasensitive sensor for the detection of ENR in biological fluids as well as in environmental samples, and this may be due to the aggregation effect that occurs between GdSn-PQ nanosheets and ENR.

### 3.7 Mechanism for ENR Detection

**Figure 9:**
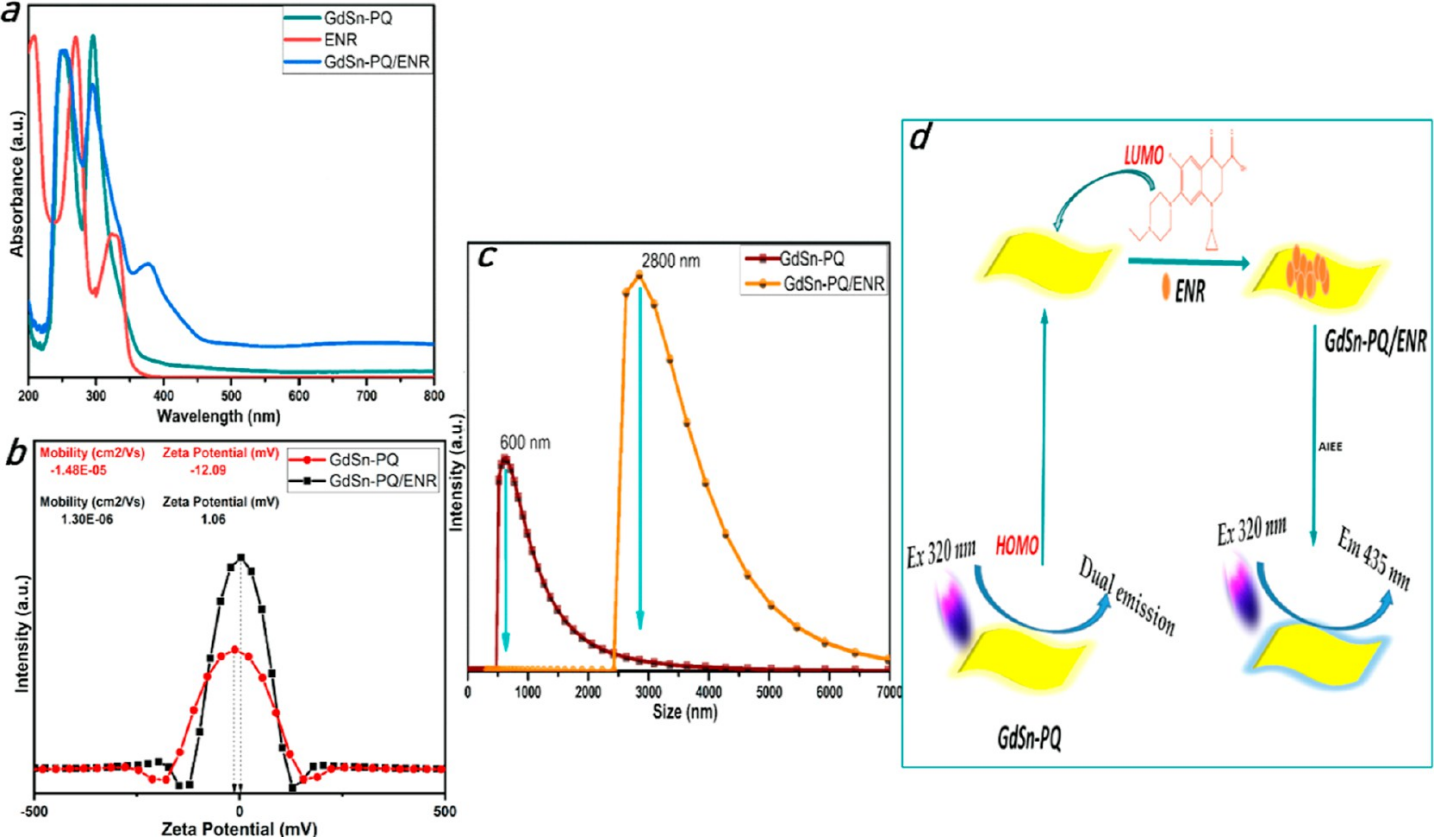
GdSn-PQ and ENR interaction mechanism studies: (a) UV absorption spectra, (b) zeta potential studies, (c) DLS size comparison, and (d) proposed diagram showing the possible interaction mechanism.

It is interesting to note that with the addition of ENR in this study, the GdSn-PQ fluorescence intensity steadily increased with a significant change in wavelength. The change in wavelength is due to the dual emission behavior of GdSn-PQ nanosheets, where one peak shows a drastic decrease in wavelength and the other shifts with the increment in intensity gradually. It is probable that the presence of extremely electron-rich metals like Gd will induce the spin states of Sn metal to delocalize, increasing the Sn electron density and enhancing the adsorption properties of nanosheets. In order to conveniently transport these electrons from GdSn-PQ to the LUMO of ENR and generate AIEE effect.^12^ UV–vis absorption spectra, atomic force microscopy (AFM), zeta potential, Raman, and DLS of GdSn-PQ were recorded before and after the addition of ENR in order to better understand the probable fluorescence process. The absorption peak of the mixture (GdSn-PQ/ENR), as shown in Figure 9a, includes all of the peaks present in the pure GdSn-PQ and ENR. Additionally, the peak at 290 nm is reduced from that of pure GdSn-PQ in aqueous solution when ENR is added, and the peak at 325 nm from GdSn-PQ that shifted to 370 nm can be responsible for the shift in fluorescence, showing the adsorption of ENR on GdSn-PQ. As can be seen in Figure 9b, the zeta potential decreased with the addition of ENR from −15.08 to 1.23 mV as a result of the interaction between GdSn-PQ and ENR and shows the adsorption of ENR on nanosheets.^12,58^ The AFM image for GdSn-PQ is shown in Figure S8a, having an elevated microscopy signal at the maximum of 4.7 nm (Figure S8b). This is acknowledged as few-layer thick nanosheets, with a nearly 0.9 nm single-layer thickness. Figure S8c shows the adsorption on the top surface of our GdSn-PQ following ENR interaction. The increase in the atomic probe height profile that reaches a maximum of ∼36 nm with the analyte is evident (Figure S8d). The increase in the atomic height can thus be attributed to the surface adsorption of ENR on GdSn-PQ nanosheets; this interaction is thus selective to the surface of nanosheets and achieves optimized sensing capabilities. Additionally, Figure 9c shows the change in size of GdSn-PQ nanosheets after the addition of ENR. The difference between the hydrated particle sizes of GdSn-PQ (0.6 μm) and GdSn-PQ/ENR (2.8 μm) shows aggregation when ENR is added. The intensity of the fluorescence emission is increased, and the nonradiative transitions of GdSn-PQ are reduced as a result of the aggregation phenomenon, which limits the intramolecular motion (vibration and rotation) of GdSn-PQ.^59^ Consequently, AIEE is a potential fluorescence enhancement mechanism, as shown in Figure 9d.

## 4 Conclusions

The 2D GdSn-PQ nanosheets with exceptional fluorescence properties were developed using a pentacene derivative as a precursor, and they have been successfully shown to be efficient probes for detecting ENR in biological and environmental samples. The GdSn-PQ nanosheets were first created or synthesized in this study by using the time-saving ultrasonication process. Gd and Sn metals, along with an organic scaffold, have also been introduced for the first time. GdSn-PQ nanosheets with ENR demonstrated exceptional performance with the detection limit of 0.10 nM in a broad linear range from 5 to 90 nM owing to the AIEE effect. The designed sensor was also tested in biological fluids (including human urine and blood serum) and environmental samples (seawater and tap water) for the quantitative analysis of ENR, and the estimated results were outstanding with a recovery rate of more than 100%. This study reveals that dual-emission has a wide range of potential applications in enhancing the detection sensitivity of GdSn-PQ for ENR, offers a creative concept for creating an effective sensor, and furthers its widespread use.

## Supporting Information Available

Controlled experiments for PL calibration based on different Gd:Sn ratios in PQ solution; fluorescence comparison of PQ with GdSn-PQ nanosheets; TEM images of GdSn-PQ nanosheets; C1s XPS spectrum for GdSn-PQ nanosheets; chemical structures of all antibiotics used in this study; biological and environmental real samples’ fluorescence studies; AFM analysis; and comparison of the present work with other reported studies detecting ENR (PDF)
