# Supplementary material for: Pentacenequinone-Modulated 2D GdSn-PQ Nanosheets as a Fluorescent Probe for the Detection of Enrofloxacin in Biological and Environmental Samples
Source: ACS Appl Mater Interfaces. 2024 May 16;16(21):27028–39. doi: 10.1021/acsami.4c00277 (PMC11145593; doi:10.1021/acsami.4c00277)
Supplement: Supplementary file 1 — am4c00277_si_001.pdf [file am4c00277_si_001.pdf]

Supporting information

Pentacenequinone-Modulated 2D GdSn-PQ Nanosheets as a Fluorescent Probe for the Detection of Enrofloxacin in Biological and Environmental Samples

Deepak Dabur[†, ‡], Priyanka Rana[‡], Hui-Fen Wu*[†, ‡, §, ||, ⊥, #, △]

[†]International PhD Program for Science, National Sun Yat-Sen University, Kaohsiung, 80424, Taiwan

[‡]Department of Chemistry, National Sun Yat-Sen University, Kaohsiung, 70, Lien-Hai Road, Kaohsiung, 80424, Taiwan

[§]School of Pharmacy, College of Pharmacy, Kaohsiung Medical University, Kaohsiung, 807, Taiwan

[||]Institute of Medical Science and Technology, College of Medicine, National Sun Yat-Sen University, Kaohsiung, 80424, Taiwan

[⊥]Institute of Precision Medicine, College of Medicine, National Sun Yat-Sen University, Kaohsiung, 80424, Taiwan

[#]School of Medicine, College of Medicine, National Sun Yat-Sen University, Kaohsiung, 80424, Taiwan

[△]Institute of Bio Pharmaceutical Science, National Sun Yat-Sen University, Kaohsiung 80424, Taiwan

*Corresponding author, Phone: +886-7-5252000-3955; Fax: +886-7-5253909

Email: hwu@faculty.nsysu.edu.tw (Prof H.-F. Wu)

Reagents

All of the solvents used in the studies were HPLC-grade solvents that were newly acquired from Sigma Aldrich in the United States. N-Methylcyclohexylamine (98%) and 2-Methyl 1,4-Naphthoquinone (98.5-101%) were acquired from DUKSON Pure Chemicals in South Korea, while HPLC grade Ethanol and Chloroform were purchased from JT Baker. purchased from SIGMA ALDRICH and ACROS ORGANICS. Gadolinium oxide (Gd_2O_3) and tin chloride ($SnCl_2$) were bought from Alfa Aesar in the United States. The following antibiotics, among others, were obtained from Sigma Aldrich, United States: Enrofloxacin, ciprofloxacin, ampicillin, spectinomycin, and lomefloxacin.

Instrumentations

Thermo Scientific's UV-Vis absorption spectroscopy EVOLUTION 201 (U.S.) was utilized to measure the optical characteristics, and Hitachi Fluorescence Spectrophotometer F-2700 (Hitachi, Japan) was employed to record the results of the fluorescence spectroscopy. Using a JOEL JEM2100, Japan, and HAADAF transmission electron microscope (TEM), the structural properties of the nanomaterials were investigated. XPS measurements were made using the Auger electron spectroscopy (JEOL, Japan) and XPS calculations were made using the DLS ELSZ-2000 (Otsuka Electronic, Japan) and Raman measurements were made by depositing the self-assembled samples as prepared on the glass substrate using setup = 633nm laser (JOBIN-YVON T64000), USA.

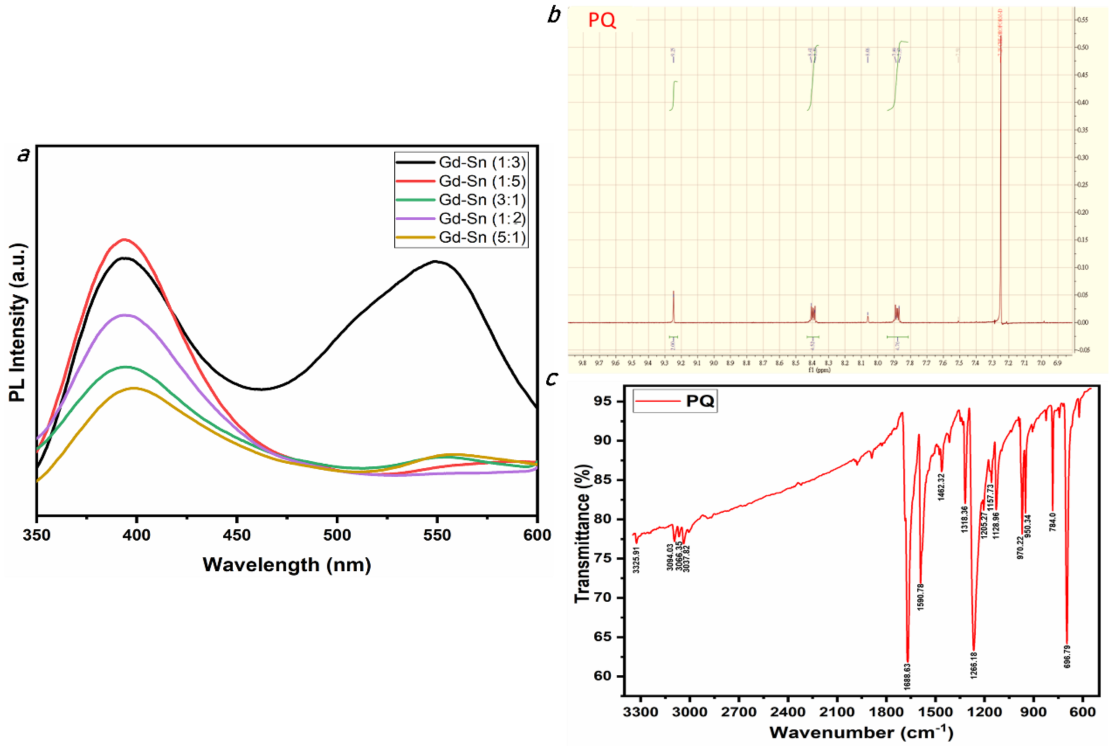

Figure S1. (a) controlled experiments for PL calibration based on different Gd: Sn ratio in PQ solution. Structural characterization of PQ **(b)** NMR **(c)** FT-IR.

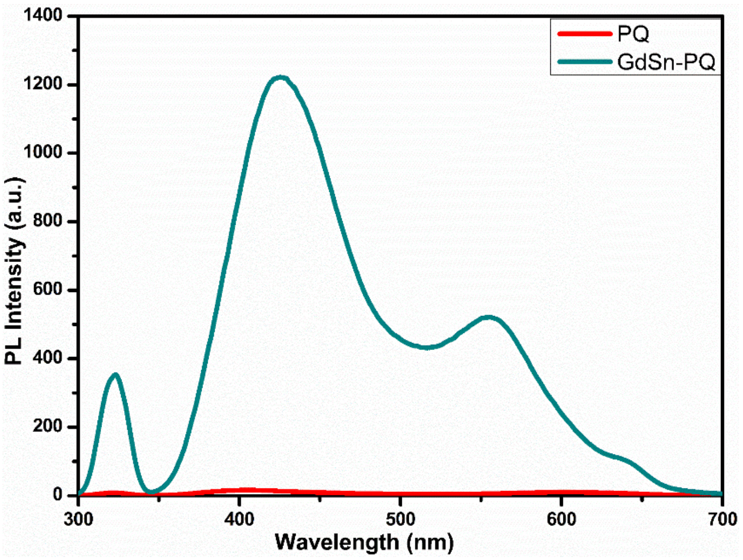

Figure S2. Fluorescence comparison of PQ (Precursor) with GdSn-PQ nanosheets.

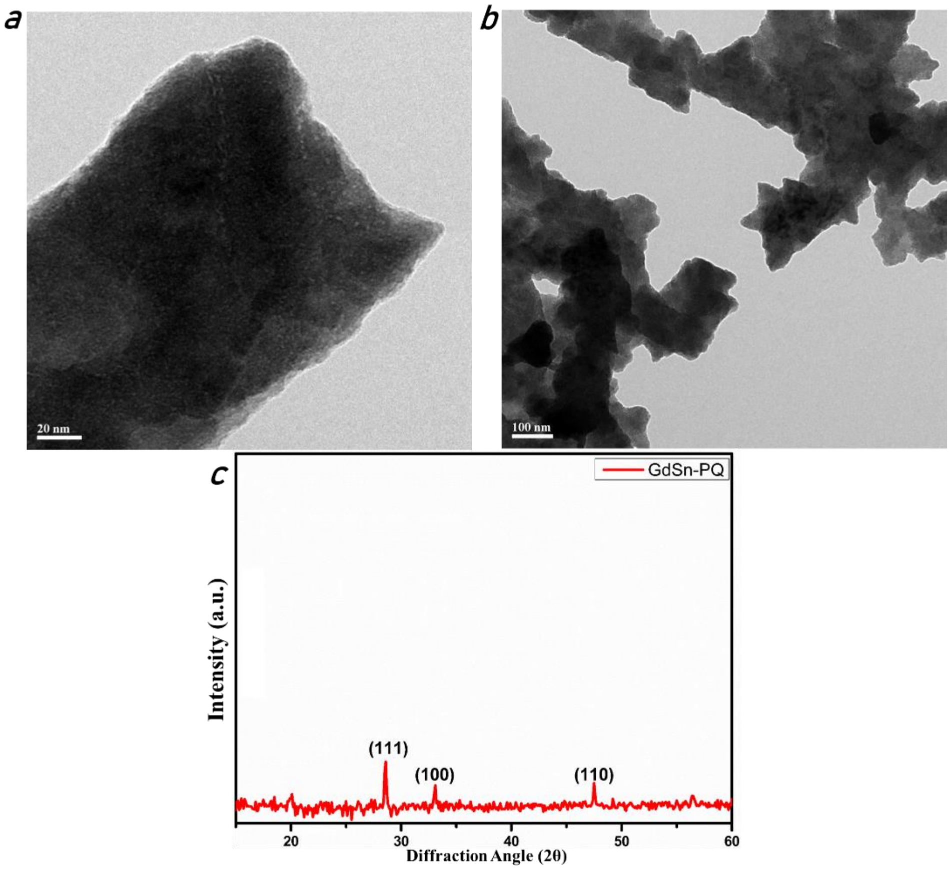

Figure S3. TEM images of GdSn-PQ nanosheets on **(a)** 20 nm and**, (b)** 100 nm scale. **(c)** XRD spectrum for GdSn-PQ nanosheets.

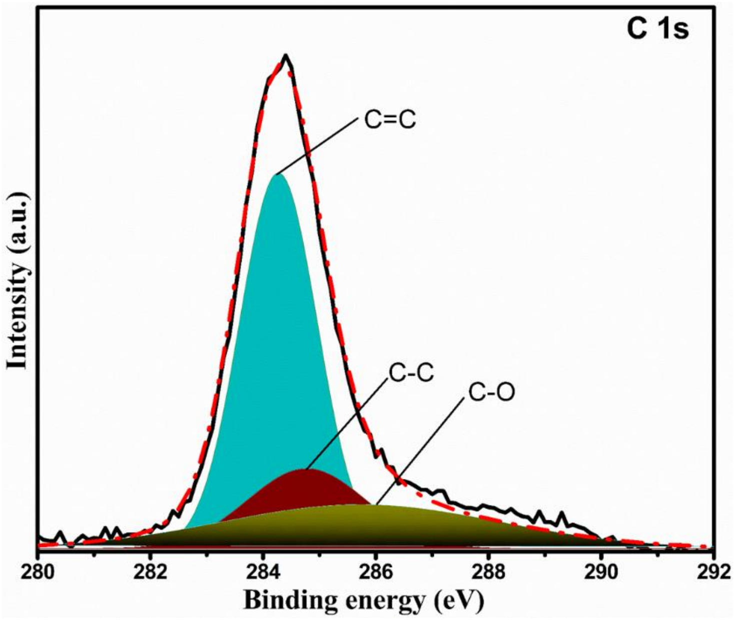

Figure S4. C1s XPS spectrum for GdSn-PQ nanosheets.

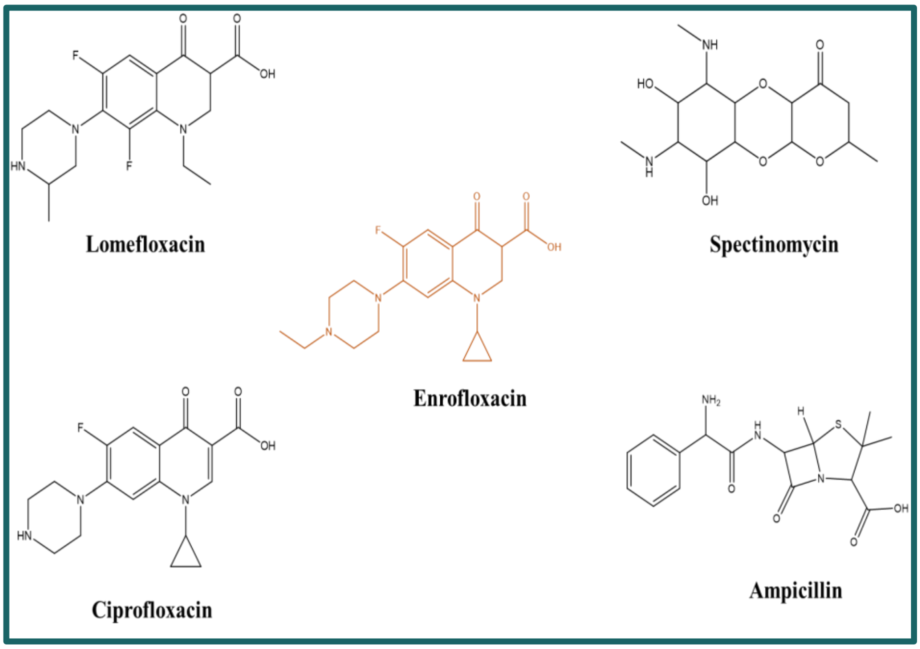

Figure S5. Chemical structure of all antibiotics used in this study.

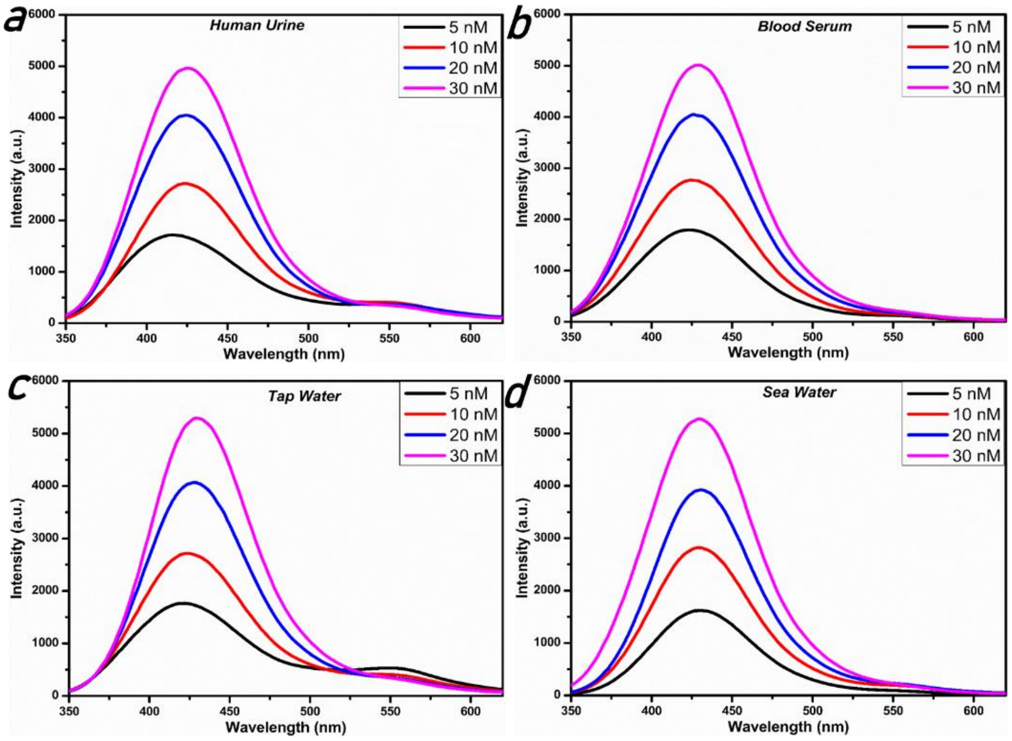

Figure S6. Biological and environmental real samples fluorescence studies **(a)** human urine **(b)** blood serum **(c)** Tap water **(d)** Sea water.

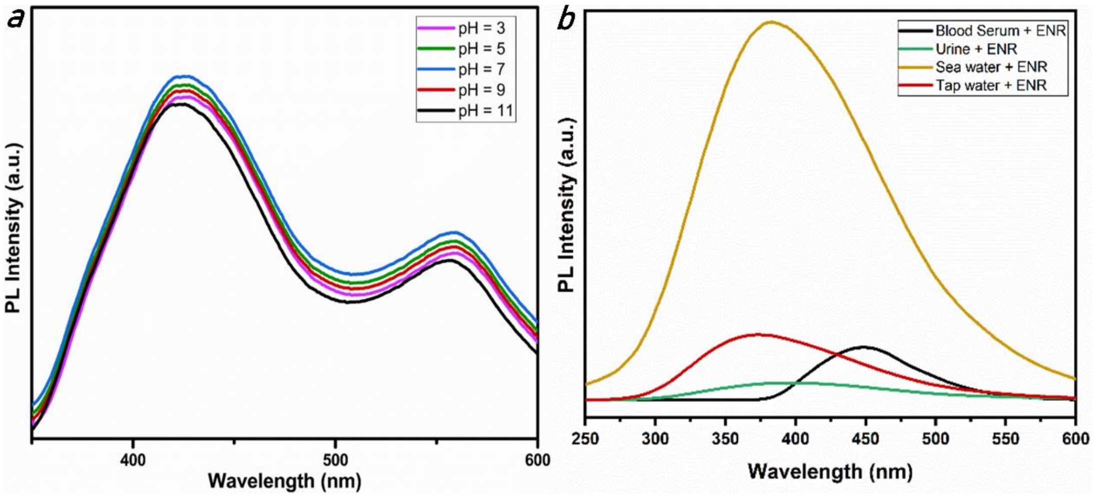

Figure S7. (a) pH based PL study of GdSn-PQ nanosheets **(b)** PL study of pure ENR in different matrices.

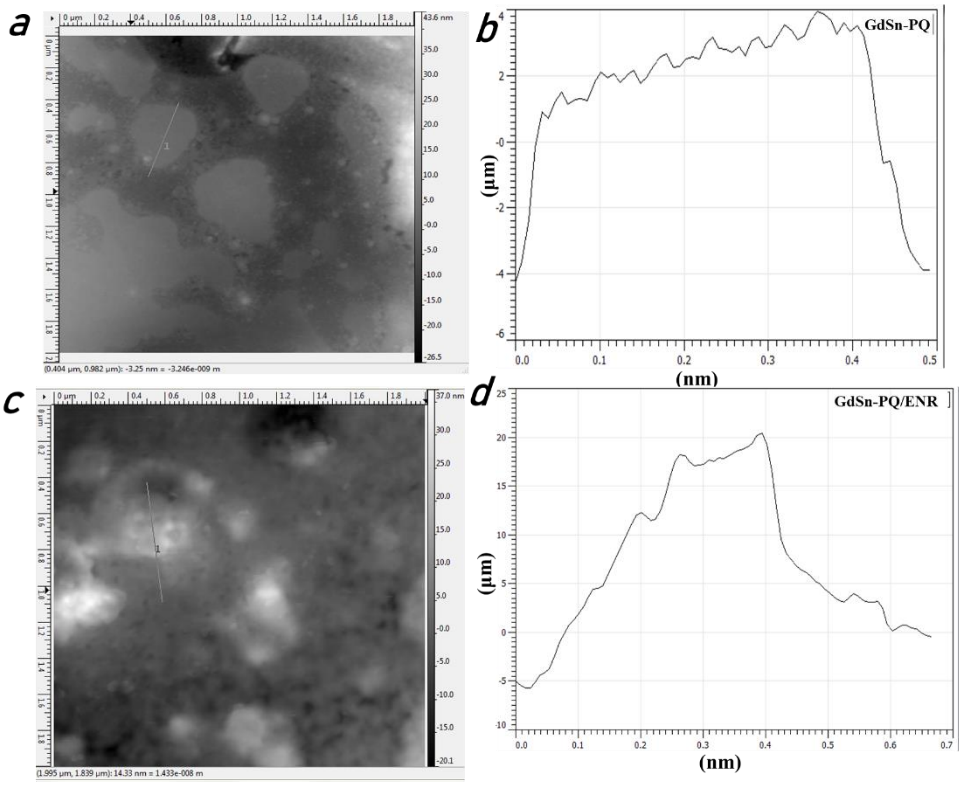

Figure S8. Atomic Force Microscopy analysis **(a)** AFM height image and **(b)** corresponding height profile of GdSn-PQ nanosheets. **(c)** AFM height image and **(d)** corresponding height profile of GdSn-PQ nanosheets showing after the addition of ENR.

Table S1. Comparison of the present work with other reported studies detecting ENR.

Material	Method	mechanism	Linear range	LOD	Real sample	Ref.
HPLC-FLD	HPLC	-	30-100 μg/kg	6.57 ug/kg	maize	1
Pf-CDs	Fluorescence	AIEE with no shift	0.20–100 μM	76 nM	Tap water	2
AgNP	SERS	Quenching	1.0–200 nmol/L	10^{-11} mol/L	-	3
CoNCs	ECl	Quenching	0.1 nM-1 μM	27 pM	Urine and water	4
AuNP	ICA	-	12–80 ng/mL	0.42 ng/ml	milk	5
AuNP and QD	FQ-ICA	Quenching		0.25 μg/L	Animal products	6
AgNP and carbon dot				0.1 μg/L		
N-CDs-Cu^{2+}	Fluorescence	Turn On	1-15 μg·mL^{-1}	0.16 μg·mL^{-1}	Water	7
**GdSn-PQ Nanosheets**	**Fluorescence**	**AIEE with the shift**	**5-90 nM**	**0.1 nM**	**Biological and environmental**	**This work**

LOD calculations:

Intercept 2315.28

Slope 69.50

LOD (ENR): 3.3 x SD/S

SD= Intercept/1000 = 2315.28/1000 = 2.315

LOD (ENR)= 3 x 2.315/69.50 = **0.10 nM**

References

(1) Brito, J.; Bernardoni, V.; da Silva, T. M.; Ramos, L. S.; Gomes, M. P.; Assis, D. Development and Validation of a Rapid and Reliable HPLC-FLD Method for the Quantification of Ciprofloxacin and Enrofloxacin Residues in Zea mays. *Journal of the Brazilian Chemical Society* **2022**, *33*, 128-134.

(2) Tang, X.; Yu, J.; Ye, H.; Zhao, L. A Novel Carbon Dots Synthesized based on Easily Accessible Biological Matrix for the Detection of Enrofloxacin Residues. *Microchemical Journal* **2023**, *190*, 108690.

(3) Li, H.; Wang, M.; Shen, X.; Liu, S.; Wang, Y.; Li, Y.; Wang, Q.; Che, G. Rapid and Sensitive Detection of Enrofloxacin Hydrochloride based on Surface Enhanced Raman Scattering-Active Flexible Membrane Assemblies of Ag Nanoparticles. *Journal of Environmental Management* **2019**, *249*, 109387.

(4) Wang, D.; Jiang, S.; Liang, Y.; Wang, X.; Zhuang, X.; Tian, C.; Luan, F.; Chen, L. Selective Detection of Enrofloxacin in Biological and Environmental Samples using a Molecularly Imprinted Electrochemiluminescence Sensor based on Functionalized Copper Nanoclusters. *Talanta* **2022**, *236*, 122835.

(5) Han, M.; Gong, L.; Wang, J.; Zhang, X.; Jin, Y.; Zhao, R.; Yang, C.; He, L.; Feng, X.; Chen, Y. An Octuplex Lateral Flow Immunoassay for Rapid Detection of Antibiotic Residues, Aflatoxin M1 and Melamine in Milk. *Sensors and Actuators B: Chemical* **2019**, *292*, 94-104.

(6) Li, S.; Wang, Y.; Mu, X.; Sheng, W.; Wang, J.; Wang, S. Two Ffluorescence Quenching Immunochromatographic Assays based on Carbon Dots and Quantum Dots as Donor Probes for the Determination of Enrofloxacin. *Analytical Methods* **2019**, *11* (18), 2378-2384.

(7) Guo, X.; Zhang, L.; Wang, Z.; Sun, Y.; Liu, Q.; Dong, W.; Hao, A. Fluorescent Carbon Dots based Sensing System for Detection of Enrofloxacin in Water Solutions. *Spectrochimica Acta Part A: Molecular and Biomolecular Spectroscopy* **2019**, *219*, 15-22.
